# Recent Advances in Ophthalmic Drug Delivery

**DOI:** 10.3390/pharmaceutics14102075

**Published:** 2022-09-29

**Authors:** Anuj Chauhan, Laurence Fitzhenry, Ana Paula Serro

**Affiliations:** 1 Chemical and Biological Engineering, Colorado School of Mines, Golden, CO 80401, USA; 2 Ocular Therapeutics Research Group (OTRG), Pharmaceutical and Molecular Biotechnology Research Centre, Department of Science, South East Technological University, X91 K0EK Waterford, Ireland; 3 Centro de Química Estrutural, Institute of Molecular Sciences, Instituto Superior Técnico, Universidade de Lisboa, Av. Rovisco Pais, 1049-001 Lisboa, Portugal

Due to population aging and to the increasing prevalence of diseases such as diabetes, chronic eye disorders such as glaucoma, age-related macular degeneration, and diabetic retinopathy have increased significantly, becoming responsible for a high percentage of blindness and vision impairment cases at a global level. Recent advances in pharmacotherapy have greatly improved their prognosis. However, the efficacy and safety of the treatments currently available are still a concern. Ophthalmic drug delivery to treat these, and other, diseases is a challenging task due to the various anatomic and physiological barriers that limit the drug entry both in the anterior and the posterior segments of the eye. Designing an ideal drug delivery system that leads to enhanced drug bioavailability compared with conventional forms of treatment (typically eyedrops and intraocular injections) ensures a controlled drug release at the target site, and helps to overcome the various ocular barriers; such an approach has been pursued by a number of researchers. This Special Issue presents some of the most recent cutting-edge research in ophthalmic drug delivery, highlighting, among other subjects, the possibility of using ophthalmic lenses as drug delivery vehicles or the inclusion of nanostructures in the delivery systems to control the drug release. It gathers nine research articles and five reviews that illustrate the high standard of research carried out by teams from 11 different countries and their efforts to develop new strategies to treat different ocular diseases.

In the first paper, Sosnik et al. [1] produced and characterized mucoadhesive-mixed polymeric micelles (PMs) made of chitosan (CS) and poly(vinyl alcohol) backbones graft-hydrophobized with short poly(methyl methacrylate) blocks, and used them to encapsulate cannabidiol (CBD), an anti-inflammatory cannabinoid. They presented a spherical morphology, sizes of 100–200 nm, and a CBD loading capacity of 20% *w/w*. Experiments with corneal epithelial cells were performed to demonstrate their biocompatibility and ability to permeate cell monolayers both under liquid–liquid and air–liquid conditions, highlighting the potential of such polymeric nanocarriers for ocular drug delivery.

Ghosh et al. [2] evaluated the pharmacological efficacy of xanthohumol, a naturally occurring prenylated chalconoid, in preclinical models for dry eye disease. They found that it prevented *tert*-butyl-hydroperoxide-induced loss of cell viability in human corneal epithelial cells in a dose-dependent manner, and resulted in a significant increase in expression of the transcription factor nuclear factor erythroid 2-related factor 2 (Nrf2), the master regulator of phase II endogenous antioxidant enzymes. Poly(lactic-co-glycolic acid) nanoparticles (PLGA NP) encapsulating xanthohumol were cytoprotective against oxidative stress in vitro, and significantly reduced ocular surface damage and oxidative stress-associated DNA damage in corneal epithelial cells in the mouse-desiccating stress/scopolamine model for dry eye disease in vivo. The authors concluded that the drug-loaded PLGA NPs constitute a safe and efficacious drug delivery vehicle for hydrophobic small molecules to the ocular surface.

The treatment of uveal melanoma (UM) was the focus of the work of Xie et al. [3]. They produced a bioinspired in situ gelling hydrogel system composed of naturally occurring biopolymer collagen and hyaluronic acid. Curcumin with anti-cancer progression and anti-metastasis effects was chosen as model drug to be delivered. The developed system gelled at 37 °C within two minutes, demonstrated excellent biocompatibility and slow degradation, and was able to sustain the drug release up to four weeks. Moreover, it showed effective inhibition of human UM cell proliferation, demonstrating a great potential for the treatment of the rare and devastating intraocular cancer.

Torres Luna et al. [4] investigated the use of fatty acids in silicone hydrogel contact lenses for prolonging the release of cationic drugs. They observed that the drug-release kinetics was dependent on the carbon chain length of the fatty acid loaded in the lens: the higher the chain length, the higher the uptake and the release duration of ketotifen fumarate (KTF) and tetracaine hydrochloride (THCL). They also found that the release duration of KTF and THCL decreased with increasing ionic strength of the release medium, and when the pH dropped from 7.4 to 5.5. Finally, the use of boundary charges at the polymer–pore interfaces of a contact lens to enhance drug partition and extend its release was further confirmed by loading cationic phytosphingosine in contact lenses to attract an anionic drug.

Kumari et al. [5] developed at a pilot scale an innovative ophthalmic formulation based on a dexamethasone-encapsulated cholesterol–Labrafac™ lipophile nanostructured lipid carrier (NLC), to be used in eyedrops for the treatment of dry eye disease (DED) and its symptoms. Using particles of 19.51 ± 0.5 nm, it was achieved an encapsulation efficiency of 99.6 ± 0.5%, a PDI of 0.08, and an extended stability of 6 months at 4 °C. The formulation presented high tolerability and internalization capacity for human corneal epithelial cells, demonstrating a similar behavior in ex vivo porcine cornea studies. ELISA allowed to study the impact of the formulation on a range of inflammatory biomarkers. A 5-fold reduction in TNF-α production over dexamethasone solution alone was observed, with similar results for MMP-9 and IL-6. The ease of formulation, scalability, performance, and biomarker assays suggest that this NLC formulation could be a viable option for the topical treatment of DED.

To understand how the asymmetry of the corneal tissue can affect the drug diffusion when dealing with topical drug formulations or intraocular drug-delivery systems, Toffoletto et al. [6] studied the permeability of two commonly administered anti-inflammatory drugs (bromfenac sodium and dexamethasone sodium) using Franz diffusion cells and porcine corneas in both inward and outward configurations. The drug accumulation in the cornea was significantly higher in the studies performed in the outward direction, which can prolong the therapeutic effect of intraocular drug-release systems. Moreover, a higher permeability coefficient was obtained for bromfenac in the outward direction, but no differences were detected for dexamethasone in the two directions.

In another work, Vivero-Lopez et al. [7] developed contact lenses (CLs) with antibiofilm and antioxidant properties by copolymerization with the antifouling monomer 2-methacryloyloxyethyl phosphorylcholine (MPC) and load them with the antioxidant resveratrol which also has antibacterial activity. Both poly(hydroxyethyl methacrylate) (HEMA) and silicone hydrogels were prepared without and with MPC (various concentrations till 101 mM), exhibiting adequate physical properties to be used in CLs. Moreover, they were nonirritant. Silicone hydrogels demonstrated a higher affinity for resveratrol. The drug released did not photodegrade, keeping its antioxidant activity. Ex vivo tests allowed to conclude that it accumulated both in cornea and sclera but did not cross these tissues. The authors found that the antibacterial capability of resveratrol was dependent on its concentration and that lysozyme adsorbed preferentially relatively to albumin, which may also contribute to the antimicrobial activity. Overall, the designed hydrogels could host therapeutically relevant amounts of resveratrol to be sustainedly released on the eye, providing antibiofilm and antioxidant performance.

Kwon et al. [8] showed that adenosine tetraphosphate (ATP) significantly improved the transport and permeation of a polymeric nanocarrier across the retina via intravitreal administration. Chitosan-functionalized, pluronic-based nanocarrier, which can deliver various biomolecules efficiently, was used as a polymeric nanocarrier. Mixing with ATP enhanced the diffusion of the nanocarrier in the vitreous humor by reducing the electrostatic interaction between this and negatively charged glycosaminoglycans in the vitreous humor. Furthermore, it also favored the penetration of the nanocarrier across the whole retina, leading to an increase in the transport of the nanocarrier across the retina of ≈9 times, as well as spreading it throughout the whole retina upon intravitreal administration in a mouse model. These effects were only observed with ATP but not with GTP, supporting a mechanism of P2Y receptor-mediated tight junction disruption by ATP.

The work of Rodrigo et al. [9] demonstrated that a glaucoma treatment combining a hypotensive and neuroprotective intravitreal formulation (IF) of brimonidine–Laponite (BRI/LAP) could be monitored non-invasively using vitreoretinal interface imaging over 24 weeks. They analyzed qualitatively and quantitatively the changes in vitreous (VIT) signal intensity, expressed as a ratio of retinal pigment epithelium (RPE) intensity. Vitreous hyperreflective aggregates mixed in the VIT and tended to settle on the retinal surface. Relative intensity and aggregate size decreased over 24 weeks in treated rat eyes as the BRI/LAP IF degraded. VIT/RPE relative intensity and total aggregate area correlated with brimonidine levels measured in the eye. They concluded that OCT-derived VIT/RPE relative intensity might be a useful marker for non-invasive monitoring of BRI/LAP IF.

González-Fernández et al. [10] presented a review that highlights the most recent efforts to implement the design of experiments to produce optimized lipid-based nanocarriers intended for ophthalmic administration. A background and overview of the various possible approaches are given, serving as base to introduce the design of experiments in current nanoparticle research. According to the authors, the quality and specifications of the product can be predicted through quality-by-design (QbD) implementation in both research and industrial stages, instead of the current quality-by-testing (QbT) framework. Mathematical modelling of the expected final nanoparticle characteristics by variation of operator-controllable variables of the process can be done performing adequate statistical design-of-experiments (DoE). Such multivariate approach allows the optimization of drug delivery platforms, maximizing the understanding of the production process and reducing research costs and time.

Wan et al. [11] are authors of another review published in this Issue. It summarizes the latest preclinical and clinical progress in suprachoroidal delivery of therapeutic agents, including small molecule suspensions, polymeric entrapped small molecules, gene therapy (viral and nonviral nanoparticles), viral nanoparticle conjugates (VNCs), and cell therapy. The authors state that formulation customization is critical to achieve favorable pharmacokinetics and report promising results obtained with novel therapeutic agents (e.g., viral and nonviral gene therapy, VNCs). They consider that a continued drug delivery research and optimization, together with customized drug formulations, may answer to many unmet therapeutic needs, targeting the affected tissues with new therapies for higher efficacy, compartmentalizing therapies away from unaffected tissues for higher safety, and achieving the required durability to relieve the burden associated to current treatments.

Toffoletto et al. [12] also prepared a review reporting the latest advances in the development of therapeutic ophthalmic lenses for the treatment and/or prophylaxis of eye pathologies (i.e., glaucoma, cataract, corneal diseases, or posterior segment diseases). The authors recognize that despite the most common route of ophthalmic drug administration are eye drops, they raise compliance issues, entail significant drug wastage by lacrimation and low bioavailability due to the ocular barriers. The use of drug-eluting ophthalmic lenses revealed to be a non-invasive and patient-friendly approach for the sustained drug delivery to the eye, that can help overcoming these problems. The paper reports several examples of therapeutic contact lenses and intraocular lenses, prepared using different drug loading strategies, that were recently described in literature. An overview of the future perspectives and challenges in the field is also provided.

Rahić et al. [13] are authors of a review that reports the novel drug delivery systems developed to fight glaucoma. They explain the different approaches used for glaucoma treatment and why they shall be individually designed for each patient. Although the first-line treatment is medication therapy, due to the numerous disadvantages of conventional ophthalmic dosage forms, intensive work has been carried out aiming the development of novel drug delivery systems for the treatment of this disease. The review provides an overview of formulation solutions and strategies followed in the development of in situ gel systems, nanosystems, ocular inserts, contact lenses, collagen corneal shields, ocular implants, microneedles, and iontophoretic devices.

Finally, Castro-Balado et al. [14] presented a last review that gathers information on the most recent advances concerning the treatment for ocular cystinosis. Given the lack of investment by the pharmaceutical industry, together with the limited stability of cysteamine, new drug delivery systems (DDSs) need to be developed, allowing more comfortable dosage schedules that favor patient adherence. Promising results obtained with hydrogels, nanowafers and contact lenses, that allow a sustained cysteamine release, are presented. Moreover, different determination methods and strategies to increase the stability of the formulations are also addressed. Na overview of the challenges and advances related to new cysteamine DDSs, analytical determination methods, and possible future therapeutic alternatives for treating cystinosis is provided.

The development of new delivery systems more efficient and/or safer than the current ones will continue to be pursued by researchers all over the world, ultimately to improve patients’ health and quality of life and reduce the economic impact of ocular diseases. The editors of this Special Issue expect that its readers find the works here presented to be interesting and stimulating, and that they contribute to generate more ideas for their future work.
